# Development and evaluation of an augmented reality education program for pediatric research

**Published:** 2020-02-29

**Authors:** Alan R. Tait, Lisa Connally, Aalap Doshi, Anita Johnson, Abbey Skrzpek, Mashala Grimes, Asif Becher, Jae Eun Choi, Monica Weber

**Affiliations:** ^1^Department of Anesthesiology, Michigan Institute for Clinical and Health Research, Michigan Medicine, and ALTality Inc., Ann Arbor, MI, USA; ^2^Department of Michigan Institute for Clinical and Health Research, Michigan Medicine, and ALTality Inc., Ann Arbor, MI, USA; ^3^Department of ALTality Inc., Ann Arbor, MI, USA

**Keywords:** augmented reality, paediatrics, research

## Abstract

**Background:**

Children often have limited understanding of clinical research and what they might expect from participating in a clinical study. Studies, however, suggest that multimedia delivery of medical and research information may promote greater understanding and engagement compared with standard written approaches.

**Aim:**

This study was designed to examine the effects of a novel interactive augmented reality (AR) program on children’s understanding of clinical research.

**Methods:**

Children (ages 7-13 years) were randomized to receive the basic information about clinical research using either a printed storybook (control) or the same storybook enhanced using a video see-through AR iPad program (AR) with embedded interactive quizzes. Children were interviewed to assess their understanding of the material before (pre-test) and after (post-test) receiving either of the randomized interventions. Both parents and children completed short surveys to measure their perceptions of the information delivery.

**Results:**

Ninety-one parent/child dyads were included in the analysis. There were no differences between the control and AR children’s pre-test understanding of the research information. However, both groups demonstrated significant and similar improvements in post-test understanding. Parents of children in the AR group found the information to be of higher quality and greater clarity compared with the control group, and 91.7% of children in the AR group found the inclusion of interactive quizzes to be helpful. Both parents and children found the AR program very easy to use and 85.0 % and 71.2%, respectively, indicated that if recruited for a future study that they would prefer information delivered using some type of iPad AR program together with a discussion with the researcher.

**Conclusions:**

Results demonstrated the importance of providing children and parents with information in an easy to read and visually compelling manner. Although both groups demonstrated improved understanding, children and their parents preferred the AR program and reported a preference for receiving information using computer-based technology. Given the seemingly insurmountable challenge of keeping children and families engaged in health research related information exchange, the use of AR would appear to provide a novel and effective vehicle for enhancing children’s and parents assimilation and understanding of research (and medical) information and as a potential tool to optimize the informed consent and assent processes.

**Relevance for patients:**

This study reinforces the importance in providing information to research participants and patients in an easy-to-read and visually salient manner. Although the AR program used in this study did not result in an increased level of understanding, AR was deemed the preferred method of information delivery. It is hoped that the results of this study will serve as a platform for future studies.

## 1. Introduction

Unlike virtual reality which immerses the user in an entirely artificial environment, augmented reality (AR) allows the virtual and real worlds to coexist and interact in real-time in a manner that promotes user engagement and active participation in learning. In this process, visually salient and contextually relevant digital information can be infused into the real environment [1]. While early AR applications focused primarily on gaming, AR has gained considerable traction for use in the military, navigation, advertising, education, and medicine. By superimposing virtual anatomic details on mannequins or real patients AR has been successfully used in medicine to teaching complex surgical and nursing techniques. Recently, AR applications have been used in our institution to help children understand their therapies and to serve as a distraction technique when undergoing minor painful surgical and medical procedures, for example, blood draws, and dressing changes.

The previous studies have shown that parents and children often have difficulty understanding both the child’s role in clinical research (what it is and what it might entail), research concepts, and specific details about clinical protocols [2-6]. In response to this, there have been a number of studies showing that interactive multimodal educational programs can improve children’s and adults’ understanding of complex research and health information over traditional paper consent forms and written educational materials [7-10]. Furthermore, many parents and children have expressed a preference for computer-based multimodal formats for the presentation of such information. Although the reasons for this are likely multifactorial, it is believed that multimodal approaches work because they provide greater visual saliency, promote active participation in learning, and for many, reduce cognitive burden by message simplification [11-13]. Multimodal interactive programs appear to be particularly beneficial for children and for adults with low literacy and numeracy abilities. Given the increasing use of digital media and the natural facility of children to interact with computer-based multimodal messaging including virtual and AR technology, we believed that AR might provide an opportunity to help children better understand important clinical research concepts and their roles as potential research participants. Therefore, this study was designed to develop and evaluate an interactive AR program for clinical research education for children and parents.

## 2. Materials and Methods

This study was deemed exempt by our Institutional Review Board.

### 2.1. Content design and development

Content for the AR program was drawn from the extant literature, expert opinion, and federal guidelines for research involving children (45CFR46 Subpart D) [14]. We were particularly interested in younger children and early adolescents who are often required to provide assent for research. Based on this information, the investigators identified and reached consensus on common themes and elements deemed most critical for inclusion into the AR program. Based on this process, the following items were deemed necessary for inclusion into the program:

1. What is clinical research and why is it important for children?
2. What types of children might be asked to participate in clinical research (e.g., sick and healthy).
3. A description of who is typically involved in the decisions regarding participation in pediatric research and an understanding that participation is voluntary and can be withdrawn at any time without penalty.
4. What children might expect from participating in a clinical research study (i.e., what a clinical study might entail including types of procedures, time commitment, and potential burden).
5. The importance of knowing the risks and benefits (direct and indirect) of participation.
6. The importance of confidentiality.
7. A series of game-related interactive exercises and quizzes to promote engagement in the material and to establish a sense of understanding.

**Figure 1:**
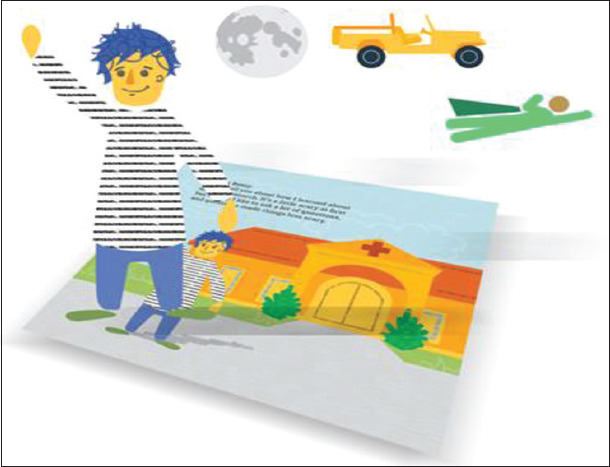
When using the AR iPad app. specific content in the storybook “comes to life” in 3-D.

The AR technology program included a storybook with generic (i.e., not study-specific) information about children’s involvement in research. The storybook introduced a gender-neutral cartoon character named “Remy” who becomes interested in the idea of participating in a research study after seeing a recruitment poster in a pediatrician’s office (Figure 1). Participants were able to select a Remy avatar as either an astronaut, explorer, or superhero each with accompanying avatar-specific background effects. Although the storybook alone provided all the basic information, when scanned with an iPad, video see-through technology initiated the overlay of 3D graphics and sound onto the storybook allowing Remy to “come to life” with action and speech. The AR program also included embedded interactive quizzes to evaluate children’s real-time understanding of the information.

The AR program was developed on a proprietary platform which leverages an Amazon Web Services back end, Vuforia for computer vision, and the Unity gaming engine. These factors make the application generally compatible for all mobile devices and AR headsets. The “assets” (images, 3D models, sounds, etc.) are called from the cloud when the application is first launched on the device and then presented when the device camera recognizes the target images in the booklet. The program was built for scalability and has been load tested to allow for hundreds of thousands of simultaneous cloud requests. In practice, this would allow a multitude of users and institutions to use the program at the same time.

Prototypes of content, characters, and voice-overs were evaluated and modified in an iterative process involving both children and adults (experts and non-experts). During this usability phase, participants were asked to verbalize their thoughts (“think aloud”) as they navigated through the program. Responses were written down verbatim and used to qualitatively assess the participants’ perceptions (likes and dislikes) of the program. Feedback from this usability testing phase was subsequently used to adjust and refine the content prior to formal evaluation.

### 2.2 Product evaluation

Child (7-13 years.) and parent dyads attending any one of our several outpatient facilities at our Children’s hospital were recruited consecutively. Baseline demographic characteristics were obtained including age and gender of the child, parental education and role (i.e., Mom, Dad, or other), and race/ethnicity. Child participants were first given a short pre-test to elicit their baseline understanding of eight core elements of clinical research (i.e., what is research, potential risks, direct benefits, indirect benefits, voluntariness, ability, and consequences of withdrawal, and who decides about participation) using a semi-structured, face-to-face interview. The responses to each question were written down verbatim by trained research assistants who were allowed to clarify questions and prompt the participants for additional information but were unable to offer any specific details. The children’s understanding of each individual core element question was scored using a 0-2 scale where 0=no understanding, 1=partial understanding or poverty of content, and 2=complete understanding. Individual item scores were subsequently combined to provide an overall score of understanding (range 0-16 where 16=complete understanding). This scoring system was based on the Deaconess Informed Consent Comprehension Test [15] and has been described previously [4,16]. Understanding was scored by individuals with no vested interest in the study and accuracy was validated by two independent researcher assistants.

Children were randomized (computer-generated) to receive information about research using either the storybook alone or the same storybook used in conjunction with the iPad AR program. Trained research assistants were available at all times to answer any questions and help children navigate through the program, if needed.

Once the subjects had either read the storybook or used the AR program, the child participants were again interviewed and scored to determine their “new” understanding of the information provided (post-test). The items in the pre-and immediate post-test interview were identical.

A survey related to the children’s perceptions of the clarity, amount of information, perceived effectiveness of the message, and their overall satisfaction with the information (i.e. AR/storybook vs. storybook alone) using 0-10 visual analog scales (where 10=high) was conducted along with the immediate post-test interview.

Parents were able to watch the program with or independently from their child but were instructed not to discuss the content or presentation of information until after the data collection was complete. Although parents were not tested for their understanding of the material per se, they were asked to complete the same survey of perceptions as did the children. At the end, parents and children were shown both the control and AR information and asked how they would prefer to receive the research information if asked to participate in a study in the future.

### 2.3. Statistical design and sample size

Sample size determination was based on data from a previous study that showed that children’s post-test understanding of clinical research concepts following exposure to a non-AR multimedia program for clinical trials was 11.65±4.1 (0-18 scale) compared with 8.85±4.1 for children who received standard text information [10]. Based on these data, we determined that we would require 45/group (parents=90 and child=90) to detect a difference in understanding of at least that size (β=20%, two-tailed) between the AR and control information.

Statistical analyses were performed using SPSS^©^ (IBM Corp, New York, v 21.0) software. Data are described as means (±SD) and medians and were analyzed using statistics for parametric (*t* tests) and non-parametric data as appropriate (e.g., Chi-square, Mann–Whitney *U*, and Wilcoxon Signed-Rank tests). Statistical significance was accepted at the 5% level (*P*<0.05).

## 3. Results

A total of 97 parent-child dyads were approached for participation in this study. Of these six were excluded due to incomplete data. Ninety-one child/parent dyads were thus included in the analysis (Control=46, AR=45). There were no differences between the control and AR groups in terms of demographics (Table 1).

**Table 1:** Demographics.

	Control (*n*=46)	AR (*n*=45)
Child’s age (years)	9.50±1.89	9.58±1.86
Child’s sex (F/M)	46.7/53.3	52.5/47.5
Parent role (mother/father)	75.6/24.4	87.2/12.8
Parent education		
Grade school	3 (6.7)	1 (2.6)
High school only	10 (22.2)	9 (23.1)
Trade school/some college	16 (35.6)	11 (28.2)
College	12 (26.7)	16 (41.0)
Graduate school	4 (8.9)	2 (5.1)
Family race/ethnicity		
White	29 (64.4)	25 (64.1)
African American	6 (13.1)	2 (5.1)
Hispanic	2 (4.4)	4 (10.3)
Native American	0 (0.0)	2 (5.1)
Asian	0 (0.0)	2 (5.1)
Other	8 (17.8)	5 (12.8)

AR: Augmented reality

There were no significant differences in baseline understanding between the two groups; both groups demonstrating poor understanding of research concepts (Table 2). There were however significant improvements in understanding following administration of both the control and AR interventions. These improvements were similar between the groups although understanding of the ability to say “no” to participation in research was significantly better in the AR group compared with the control group. Children’s understanding of the information improved with the age of the child. Older children (10-13 years based on median split) had significantly greater overall understanding of the information compared with children aged 7-9 years (11.39±2.21 vs. 9.44±2.77 out of 16, respectively, *P*<0.001). This was consistent for both the control and AR groups.

**Table 2:** Children’s Pre- and Post-Test Understanding of Research Concepts by Group.

Concept	Pre-test Con	Post-test Con	Pre-test AR	Post-test AR
Research	0.61±0.80	1.30±0.84*	0.51±0.73	1.38±0.76^†^
Risks	0.33±0.47	0.43±0.65	0.29±0.46	0.33±0.48
Direct Benefits	0.69±0.81	1.15±0.89*	0.69±0.73	1.19±0.77^†^
Indirect Benefits	0.87±0.86	1.40±0.75*	0.78±0.88	1.38±0.79^†^
Choice	1.63±0.77	1.78±0.64	1.47±0.87	1.86±0.52^†^
Say “No”	0.65±0.92	1.13±0.99*	0.71±0.95	1.63±0.77^††^
Withdraw	0.93±0.98	1.62±0.78*	1.11±0.99	1.85±0.53^†^
Who decides Total Understanding^a^	0.83±0.57 6.54±2.55	1.13±0.46* 9.93±2.60*	0.88±0.44 6.52±2.72	1.12±0.57^†^ 10.71±2.80^†^

Con: Control group: AR: Augmented Reality group. Data are mean ± SD based on scores of 0-2 where 2: Complete Understanding.

a Total understanding based on scores of 0-16.

* *P*<0.05 versus Control Pre-test value;

† *P*<0.05 versus AR Pre-test value. ^‡^*P*<0.05 verusu Control Post-test value

Both groups found the information equally helpful and clear although children in the AR group thought that there was too much information compared with the control group (Table 3). However, when split by age (7-9 vs. 10-13 years), it was found that children in the younger group were more likely to report that the amount of information in the AR program was “too much” compared with the older children (61.9% vs. 15.8%, *P*<0.005).

**Table 3:** Children’s perceptions of information delivery.

	Control	AR	*P* value
Likelihood of participation in future research based on presentation	8.02 ± 2.54	7.65 ± 2.71	0.517
Helpfulness of information			
Not at all	4 (8.9)	2 (5.1)	0.245
Somewhat helpful	32 (71.1)	23 (59.0)	
Extremely helpful	9 (20.0)	14 (35.9)	
Amount of information			
Too little	4 (8.9)	1 (2.5)	0.002
Just right	37 (82.2)	23 (57.5)	
Too much	4 (8.9)	16 (40.0)	
Clarity of information			
Not clear	5 (11.1)	4 (10.0)	0.080
Fairly clear	20 (44.4)	9 (22.5)	
Extremely clear	20 (44.4)	27 (67.5)	

AR: Augmented reality

Parents, on the other hand, perceived the AR information to be of significantly higher quality and clarity compared with the control information and were more likely to believe that the amount of information provided was “just right” (Table 4). Parents in the AR group were also significantly more likely to report a likelihood of allowing their child to participate in any future study if information was presented in AR format.

**Table 4:** Parent’s perceptions of information delivery.

	Control	AR	*P* value
Likelihood of participation in future research based on presentation	7.62±1.54	9.02±0.95	0.000
Quality of information	7.78±1.66	8.85±1.23	0.001
Ability to follow information	8.82±1.13	9.15±1.05	0.172
Helpfulness of information			
Not at all	4 (8.9)	1 (2.5)	
Somewhat helpful	30 (66.7)	24 (60.0)	0.247
Extremely helpful	11 (24.4)	15 (37.5)	
Amount of information			
Too little	17 (37.8)	3 (7.5)	
Just right	28 (62.2)	34 (85.0)	0.001
Too much	0 (0.0)	3 (7.5)	
Clarity of information			
Not clear	1 (2.2)	0 (0.0)	
Fairly clear	26 (57.8)	12 (31.6)	0.029
Extremely clear	18 (40.0)	26 (68.4)	

AR: Augmented reality. *P<0.05 vs Control. Data are mean±SD and *n* (%)

Parents were very satisfied with all aspects of the program including the interactivity and graphics (Table 5). Overall, children in the AR group found the AR program extremely easy to use (8.68±2.01 out of 10, where 10: extremely easy). The ability of children to correctly answer the embedded games/quizzes in the AR program on the first attempt ranged from 44.2 to 97.7% (Average=76.3%). Only four children (8.3%) believed that the games/quizzes were “NOT helpful.”

**Table 5:** Parents’ satisfaction with the augmented reality program.

	Satisfaction*
iPad program easy to use	8.61±1.70 (9.0)
Graphics	8.80±1.47 (9.0)
Interactivity	9.39±1.02 (10.0)
Narration	9.22±1.06 (10.0)
Sound effects	9.09±1.04 (9.0)
Quizzes/games	9.17±1.32 (10.0)
Overall satisfaction	9.20±1.14 (9.5)

* Satisfaction scores on scale of 0-10 where 10: Extremely Satisfied. Data are mean±SD (median)

Open-ended comments from both parents and children in the AR group were overall very positive. Comments included “helped child to learn that kids/adults can participate in studies;” “interactive, entertaining, helped to understand more;” “characters and reading it to you;” “like the games;” “(like) the games but hard to use book and iPad at times;” “reading it out loud;” and “listening to it/the kid’s voice.” At the end of the study, parents and children were asked how they preferred to receive information about research. Table 6 shows that both parents and children reported that they would prefer information using some type of AR program like the one used in this study together with a discussion with the researcher. This finding was consistent for both younger and older children.

**Table 6:** Children’s and parents’ preferences for information delivery.

	7-9 years*	10-13 years*	All children	Parents
Written information only	3 (9.4)	2 (7.4)	5 (8.5)	0 (0.0)
Verbal only (from investigator)	3 (9.4)	0 (0.0)	3 (5.1)	0 (0.0)
Written and verbal	0 (0.0)	4 (14.8)	4 (6.8)	7 (8.8)
iPad AR only	4 (12.5)	1 (3.7)	5 (8.5)	5 (6.3)
iPad AR and verbal	22 (68.8)	20 (74.1)	42 (71.2)	68 (85.0)

AR: Augmented Reality.

* Age based on median split

## 4. Discussion

Results from this study showed that information provided in an easy to read and visually salient manner can help children understand research concepts. The previous studies have shown that information delivered to both children and adults using computer-based multimedia results in greater participant and patient understanding of research and medical information, compared with standard written information [7-9]. This study was novel in that it expanded on the previous computer-based messaging to include AR. The observation that there were no differences in children’s understanding between the control and AR groups in this study may have, in part, been a reflection of the enhanced presentation of the information in the control booklet. Previously, we have shown that relatively simple improvements in the formatting of a standard paper consent document for a pediatric study (e.g., 8^th^ grade reading level, use of cartoons and pictographs, color, and bulleting) significantly improves child and parent understanding of the information [16,17].

However, despite the fact that no significant differences in understanding were observed between groups (other than the concept of being able to say “no”), the AR delivery of information was better received by parents and children and both reported that AR would be the preferred method of information delivery for participation in any future study. Interestingly, parents also reported that they would be more likely to consider participation in a future study for their child if presented with information using an AR medium. While participants are likely to be more obliging within the confines of a research study, keeping children and their parents engaged in potential health related information exchange in the real world are a significant challenge. In this environment, where there may be competing demands from clinical personnel and attention is fleeting, participants’ preferences for a medium play an important role in getting (and keeping) them engaged with information at hand. With this in mind, the children’s and parents’ preference for the rich, layered world of AR over the control booklet in this study is noteworthy. Almost all the children (91.7%) found the interactive games/quizzes to be helpful which supports the previous research showing that the use of exercises with corrected feedback can facilitate information retention [18,19]. Use of these interactive exercises is important in establishing a sense of real-time understanding of the information and can be used by investigators to ensure that participants understand the information at the time decisions are being made. It is thought that these interactive games facilitate understanding and provide a sense of fun by promoting active participation in learning rather than passive retention [20,21].

The potential limitations of this study are recognized. First, this study describes a single prototype intervention at one institution and, as such, may not be generalizable to all other institutions or populations. Further, we limited our sample to children between the ages of 7 and 13 years. This group was chosen because children of this age are typically asked to provide assent to participate in clinical research but often have difficulty with written information. The content of the AR program was thus designed to be appropriate to this age group. Older adolescents were therefore excluded believing that they would perhaps consider the presentation “too young” for them. We have used the term understanding throughout rather than recall. While the terms are often used interchangeably, we believe that by asking the children to describe the information in their own words provides some level of understanding.

This study reinforces the importance of presenting research information to parents and children in an easy to read and visually compelling manner. Although the AR and control groups performed equally well in promoting children’s understanding of research information, results suggest that both children and parents preferred the interactive and immersive nature of the AR technology over the more traditional written format. These results therefore support the use of enhanced information delivery to children and parents and highlight the potential promise of AR as a future technology for enhancing the assent and consent process.

### Disclosures

Jae Eun Choi is the Chief Research Officer for ALTality, Inc. which developed the AR program. Ms. Choi provided intellectual input for the project but had no involvement in subject recruitment, data collection, analysis, or interpretation of the data. None of the other investigators have any financial, commercial, or other interests in ALTality, Inc.
